# A mediastinal angiomatoid fibrous histiocytoma radically resected with the use of cardiopulmonary bypass and transection of the ascending aorta

**DOI:** 10.1093/icvts/ivae214

**Published:** 2024-12-23

**Authors:** Luis Gisli Rabelo, Christine Tolman, Brynja Jonsdottir, Tomas Gudbjartsson

**Affiliations:** Faculty of Medicine, University of Iceland, Reykjavik, Iceland; Cardiothoracic Department, Landspitali—National University Hospital, Reykjavik, Iceland; Radiology Department, Landspitali—National University Hospital, Reykjavik, Iceland; Pulmonary Department, Landspitali—National University Hospital, Reykjavik, Iceland; Faculty of Medicine, University of Iceland, Reykjavik, Iceland; Cardiothoracic Department, Landspitali—National University Hospital, Reykjavik, Iceland

**Keywords:** Angiomatoid fibrous histiocytoma, Mediastinal tumour, Surgical resection, Cardiopulmonary bypass, Transection of the ascending aorta

## Abstract

Angiomatoid fibrous histiocytoma (AFH) is a rare soft tissue tumour that rarely behaves malignant. We report a radical resection of a mediastinal angiomatoid fibrous histiocytoma, which grew invasively into the pulmonary artery wall, was adherent to the posterior aorta and close to the main stem of the left coronary artery. A transection of the aorta was performed using cardiopulmonary bypass and cardioplegic arrest for a safe and radical removal that resulted in symptom relief.

## BACKGROUND

Angiomatoid fibrous histiocytoma (AFH) is a rare, low-grade malignant soft tissue neoplasm that presents distinctive diagnostic dilemmas due to its clinical and radiological resemblance to various malignancies [1, 2]. While typically a slow-growing tumour found in the extremities of children and young adults, extra-somatic soft tissue sites of AFH, like the mediastinum, have a higher mean age of 35 at presentation and are more frequently associated with systemic symptoms such as pyrexia, anaemia and malaise [3].

Here, we describe a challenging but successful resection of a deep mediastinal AFH located behind the aortic root with invasion of the right pulmonary artery. Due to the rarity and surgical challenges of mediastinal AFH, this case highlights the need for a multidisciplinary approach, including cardiopulmonary bypass.

## CASE REPORT

**Figure 1:**
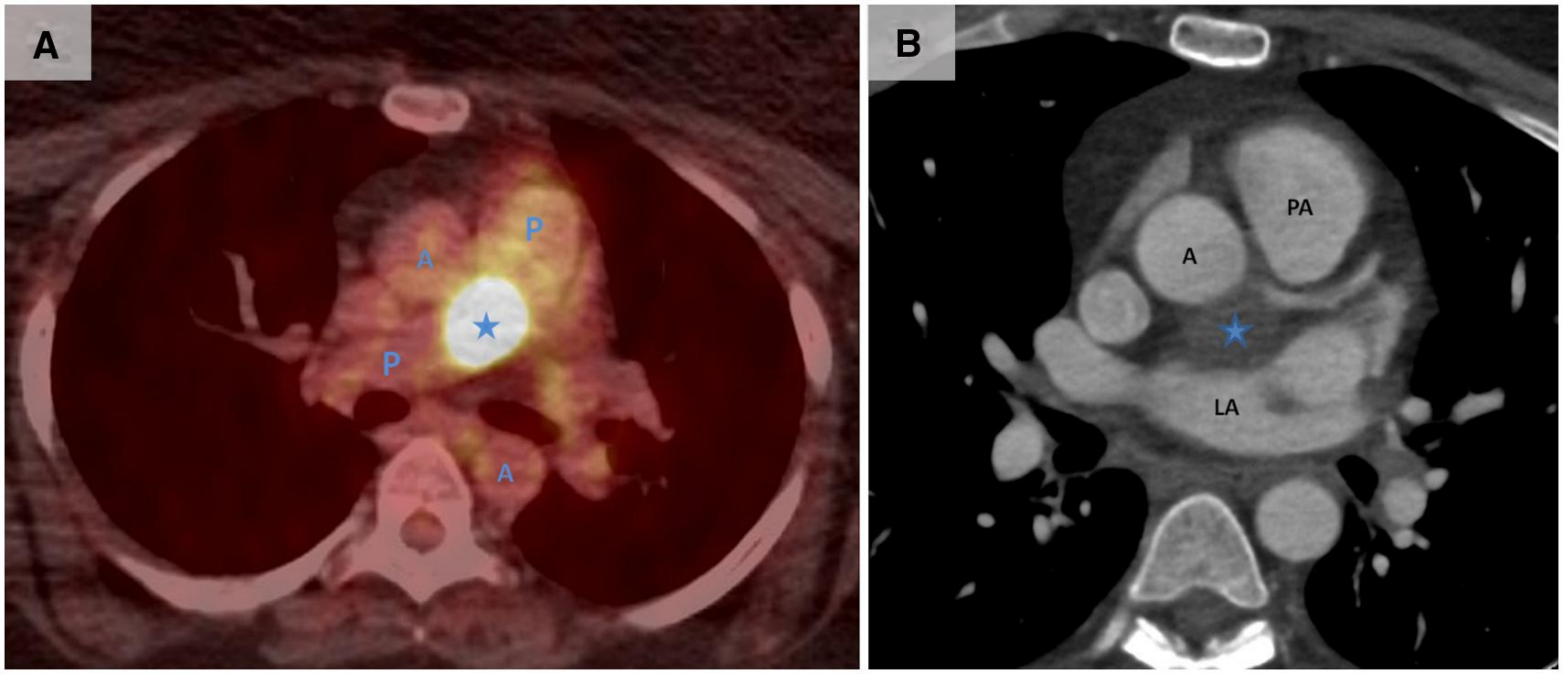
(**A**) A PET scan illustrating a hypermetabolic tumour. (**B**) Contrast-enhanced 320-slice CT scan showing the tumour (*). A: aorta; CT: computed tomography; LA: left atrium; LM: left main; P/PA: pulmonary artery; PET: positron emission tomography.

A 39-year-old woman presented with a 5-year history of progressive generalized weakness, periodic fevers, night sweats and sporadic episodes of hypertension, ultimately rendering her unable to work. Lab results revealed an elevated erythrocyte sedimentation rate of 72 mm/h (reference <20 mm/h), raising concerns for vasculitis. Subsequent positron emission tomography and computed tomography scans illuminated a hypermetabolic mass within the middle mediastinum located posterior to the aortic root, between the left atrium and the main trunk of the pulmonary artery (Fig. 1). Furthermore, this well-defined 2.4×2.7 cm mass was adjacent to the left coronary ostium and the left main stem; however, it demonstrated no enhancement on cardiac computed tomography with contrast. A magnetic resonance imaging showed that the tumour moved with the right pulmonary artery pulsation, raising suspicion of potential infiltration. Because of a high risk of bleeding from the pulmonary artery, left atrial wall and aorta, a biopsy was avoided by transthoracic puncture or mediastinoscopy. Preoperatively, neurogenic tumours such as paraganglioma or schwannoma were considered the most likely diagnosis. With the aim of symptom relief and ruling out malignancy, she was offered surgical resection, which she accepted.

A full median sternotomy was conducted, and a cryosection biopsy from the tumour raised suspicion of a neurogenic origin like schwannoma. Given the potential for malignancy and the prospect of curative resection with symptom relief, it was decided to aim for complete resection. The tumour adhered to the posterior aortic wall and aortic root, invading the right pulmonary artery. This necessitated the establishment of cardiopulmonary bypass with standard arterial cannulation of the upper ascending aorta and double-stage venous cannula via the right atrial auricle, followed by cardiac arrest using cold blood cardioplegia. The ascending aorta was transected completely 2 cm above the right coronary ostium, and the tumour was dissected from surrounding structures (Supplementary Material, Fig. S1). This included resection of the anterior wall of the right pulmonary artery to obtain complete tumour removal, which was then repaired with a 3 × 2 cm bovine patch. The cross-clamp and cardiopulmonary bypass times were 104 and 140 min, respectively. The patient was not actively cooled, the lowest temperature being 34.8°C, and intraoperative bleeding was minimal.

**Figure 2:**
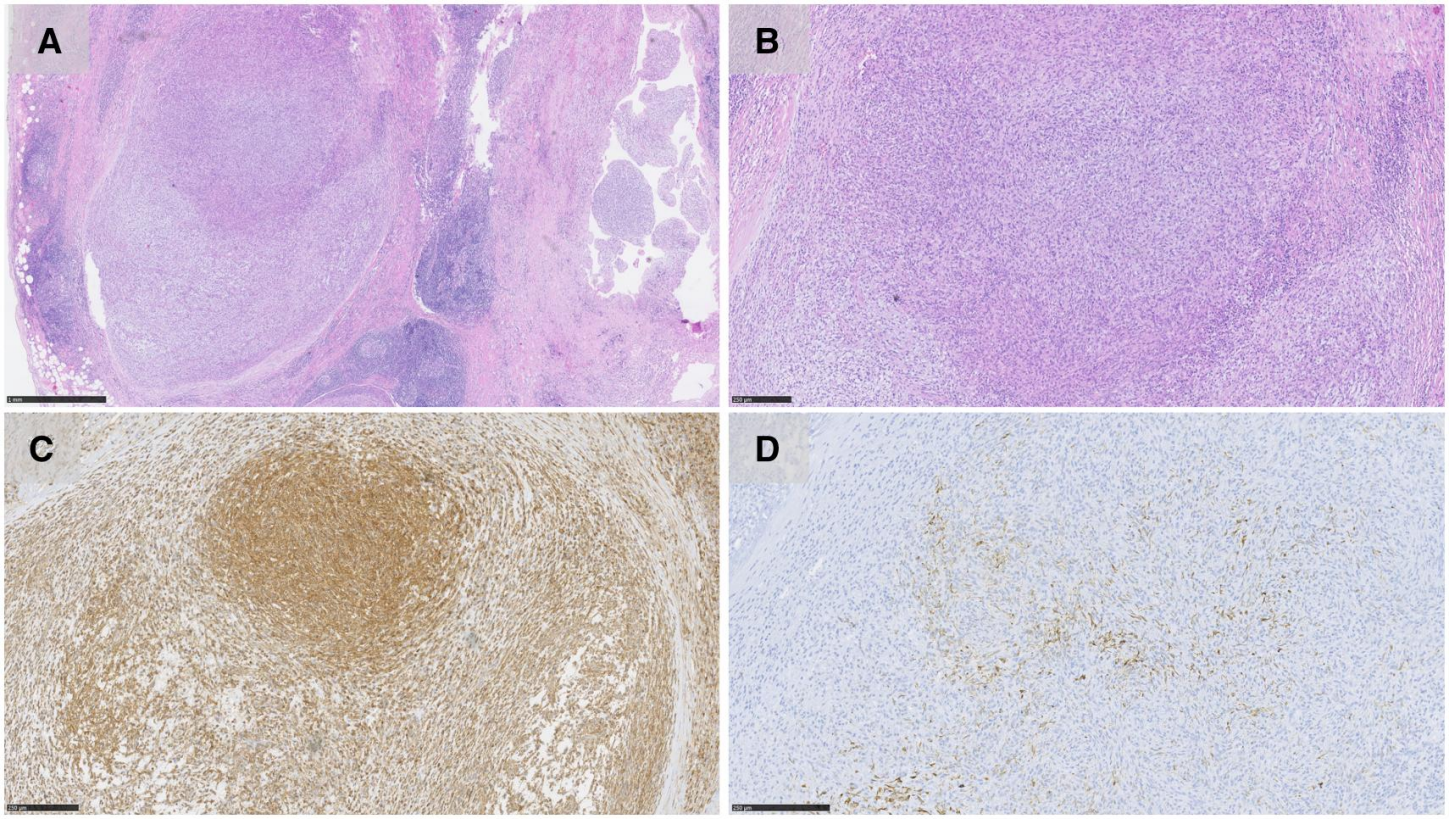
(**A**) Histology demonstrating nodular growth, lymphoplasmacytic cuffing and pseudo vascular spaces (H&E stain; 1 mm). (**B**) Spindle cell tumour with bland nuclei and syncytial pattern (H&E stain; 250 µm). (**C**) Immunoreactivity for CD99 (250 µm). (**D**) Desmin immunoreactivity (250 µm).

Postoperatively, pathologic analysis revealed a spindle cell tumour with focal nodular patterns, bland nuclei, few mitoses and necrosis absent but a fibrous pseudo-capsule associated with lymphoplasmacytic infiltrate and nerve bundle that demarcated the tumour (Fig. 2). The immunohistochemical stains were positive for CD99, EMA, ALK-protein, Desmin and S100. Negative stains were myogenin, CD34, SMA, SOX10, HMB45, and AE1/AE3. The diagnosis of AFH was then finally confirmed with a positive FISH analysis revealing rearrangement for EWSR1 (22q12). Resection margins were tumour-free, confirming a radical resection. At the 1-year follow-up, the patient continued to show resolution of symptoms, and computed tomography imaging at 16 months postoperatively confirmed no signs of recurrence. In addition, erythrocyte sedimentation rate had resolved from 72 mm/h preoperatively to 12 mm/h postoperatively. No postoperative chemo- or radiotherapy was administered.

## DISCUSSION

This case demonstrates a complex radical resection of the mediastinal AFH, resulting in symptom relief at 1-year follow-up. A conventional median sternotomy was chosen instead of a minimally invasive approach due to the tumour’s location and the risk of significant intraoperative bleeding, as has been reported in previous studies on middle mediastinal paraganglioma tumours with a similar mediastinal position [4]. In addition, the decision to use CPB was made intraoperatively to ensure a safe resection from the pulmonary artery, atrial wall, posterior aorta and left coronary main stem. Complete aortic dissection was a key step of the operation and provided optimal access to these structures for radical and controlled resection.

To our knowledge, this is the second mediastinal AFH case documented in the literature, 1st reported by Asakura *et al.* in 2001 [5]. Unlike the current case, that tumour did not invade any nearby structures but was prone to bleeding. Although AFH is characterized as a low-grade malignancy, it can demonstrate local invasive growth, including infiltration into vessel walls such as the pulmonary artery. Furthermore, radical resection of mediastinal AFH tumours seems key to alleviating symptoms and improving quality of life. Therefore, we think these tumours should be operated on, even with complex surgery, if the patient is deemed fit for such a procedure.

## Supplementary Material

ivae214_Supplementary_Data

## Data Availability

Underlying data cannot be shared publicly to protect patient privacy.
