# Supplementary material for: A mediastinal angiomatoid fibrous histiocytoma radically resected with the use of cardiopulmonary bypass and transection of the ascending aorta
Source: Interdiscip Cardiovasc Thorac Surg. 2024 Dec 23;40(1):ivae214. doi: 10.1093/icvts/ivae214 (PMC11681935; doi:10.1093/icvts/ivae214)
Supplement: ivae214_Supplementary_Data [file ivae214_supplementary_data.docx]

**Supplementary data**

**Figure S1:** The AFH tumor dissected free from the adventitia of the posterior aortic wall.
